# Iatrogenic chest tube placement within the heart following a large pneumothorax

**DOI:** 10.1016/j.radcr.2025.04.089

**Published:** 2025-05-20

**Authors:** Shervin Zoghi, Cameron L. Brock, Kwame Atsina, Yasser G. Abdelhafez, Fatma Sen, Lorenzo Nardo, Mohammad H. Madani

**Affiliations:** a School of Medicine, University of California, Davis, CA, USA; b Department of Radiology, University of California, Davis, CA, USA; c Division of Cardiovascular Medicine, Department of Internal Medicine, University of California Davis

**Keywords:** Chest tube placement, Iatrogenic, Cardiac perforation

## Abstract

This case report describes an uncommon occurrence of cardiac injury and perforation resulting from chest tube placement. The patient, a 44-year-old female, presented to the ED with severe blunt force trauma following a pedestrian motor vehicle collision. Imaging revealed a large pneumothorax and multiple fractures. Pulsatile bleeding was observed during chest tube placement, and the tube’s tip was later found on CT of the thorax to be located within the heart. Although the patient recovered by postoperative day 13 and was subsequently discharged, this case highlights the importance of performing this procedure under image-guidance, along with elucidating the critical role of serial thorough clinical evaluations and prompt imaging for early detection and management of complications.

## Introduction

Chest tube placement is a commonly utilized procedure in hospitals that is the standard of care for managing conditions such as pneumothoraces and pleural effusions. While a generally safe and well-studied procedure, adverse events, such as insertion site infections and malposition, are not uncommon complications. Although more severe complications are unlikely, this case highlights the very rare life-threatening complication-an iatrogenic cardiac perforation-during chest tube placement and the subsequent intervention required. Due to the limited literature available, the goal of this report is to emphasize the rare nature of this complication and the importance of imaging for diagnosis and intervention.

## Case presentation

A 44-year-old female was brought to the emergency department by ambulance after being struck by an automobile the previous night. EMS reported the patient was hypotensive at the scene (BP 74/60). Her medical history is significant for chronic daily methamphetamine and alcohol use, homelessness, malnutrition, cachexia, syphilis, chronic disseminated skin wounds, SVT status post ablation, and newly diagnosed HIV/AIDS.

**Fig. 1:**
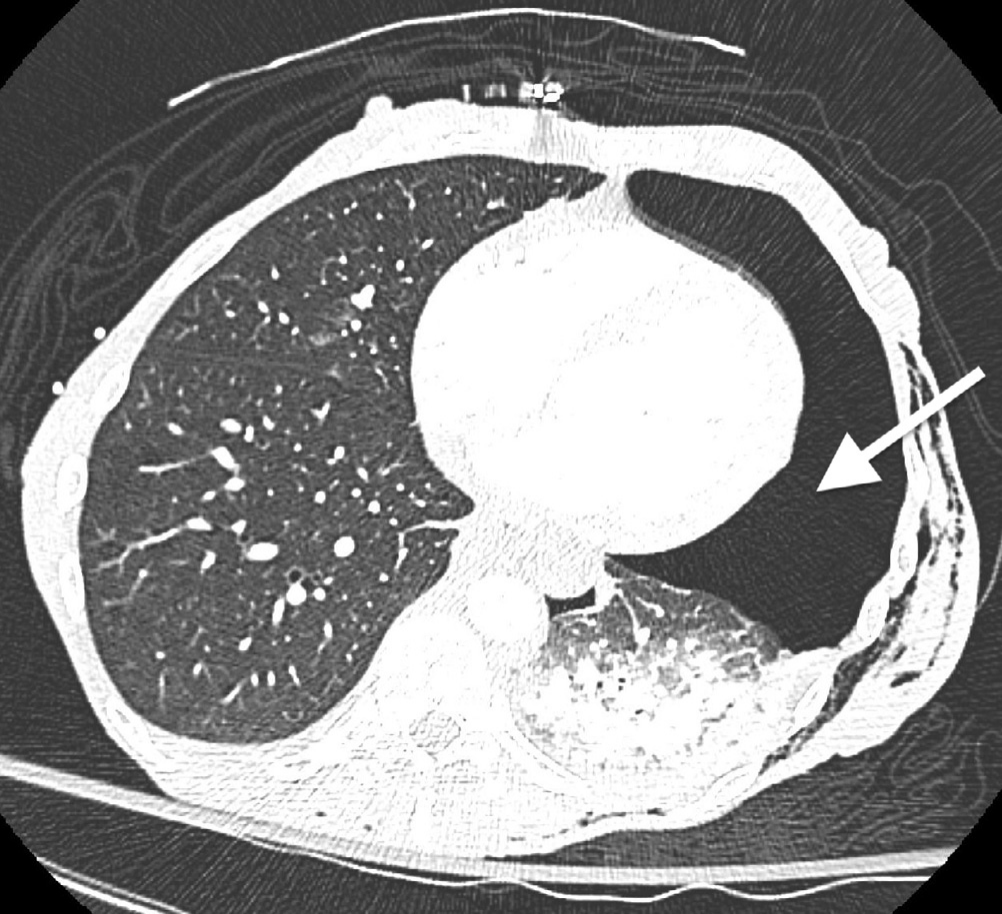
Axial CT image of the chest demonstrating large left sided pneumothorax (white arrow).

**Fig. 2:**
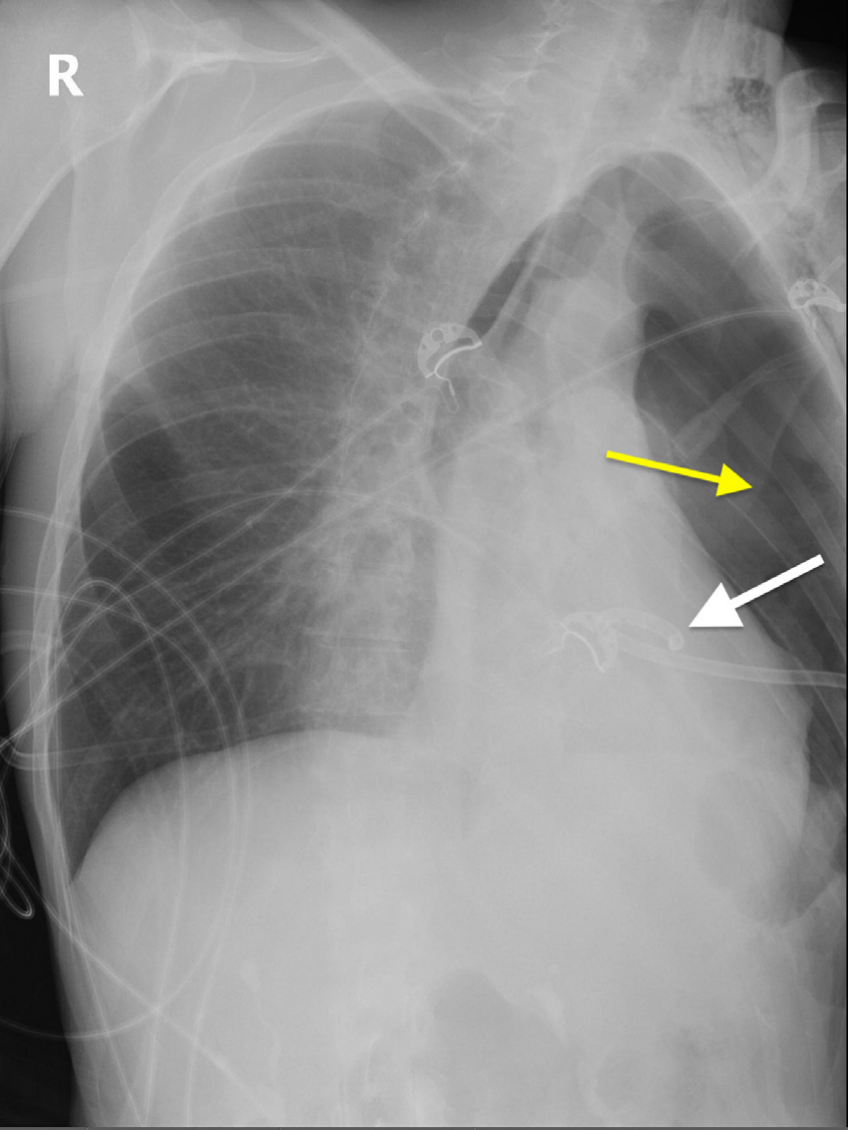
Portable 30-degrees upright chest radiograph showing interval placement of the chest tube with the tip projecting onto the cardiac silhouette (white arrow), along with the left sided pneumothorax (yellow arrow).

**Fig. 3:**
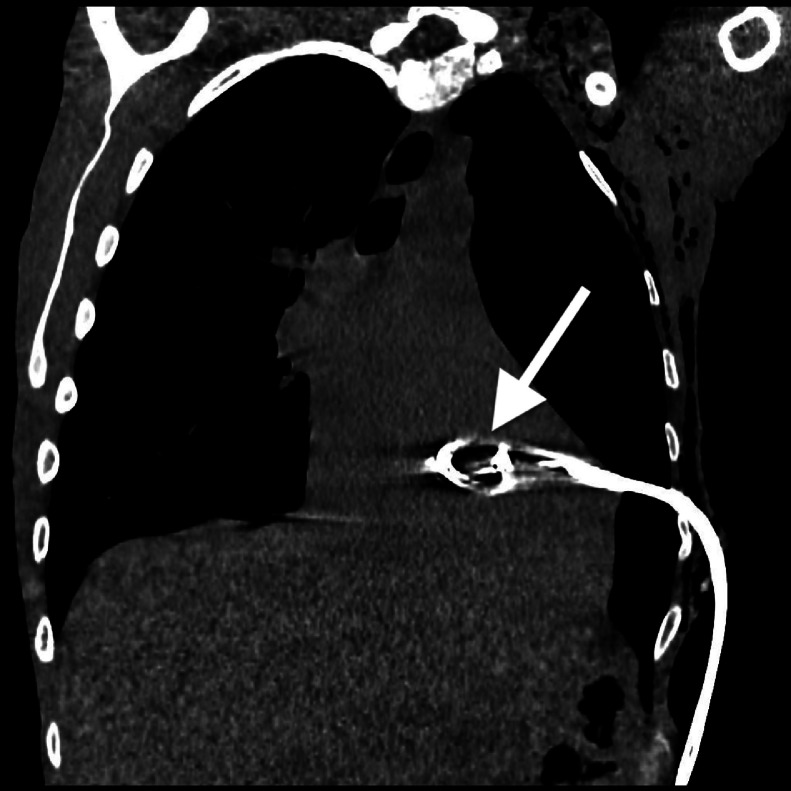
Coronal CT image of the chest (mediastinal window) showing iatrogenic intracardiac chest tube placement (white arrow).

**Fig. 4:**
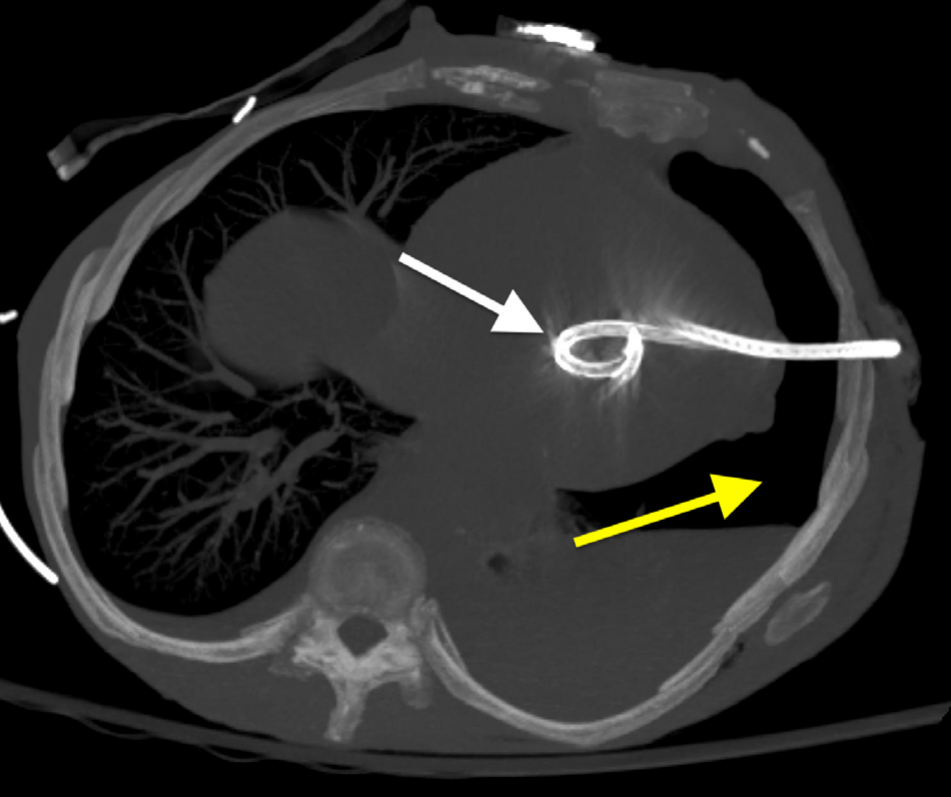
Axial MIP (Maximum Intensity Projection) CT image of the chest showing the location of the intracardiac chest tube (white arrow). CT imaging in Fig. 3, Fig. 4 played a vital role in quickly and accurately diagnosing the malpositioned chest tube and providing key information for the patient’s subsequent operation.

Upon arrival at the emergency department, her blood pressure was 113/93, pulse 63 bpm, body temperature 31.2 degrees Celsius, respiratory rate 19/min, and oxygen saturation 98% on room air. Physical examination revealed a Glasgow Coma Scale (GCS) score of 14, with neurological and abdominal exams appearing grossly unremarkable. Bilateral breath sounds and intact airway were noted, and old abrasions and scarring were visible on the right chest. An initial thoracic CT scan revealed a large, left sided pneumothorax and left lung contusion (Fig. 1). Additional findings included multi-level left sided rib, sternal, and thoracic spine fractures. After local anesthesia was injected into the subcutaneous space and periosteum, an 18-gauge needle was advanced, and air was aspirated. A J-tip guide wire was passed, and the needle was removed while the wire was secured. A skin incision was made with a scalpel, and a dilator was passed and removed. A 14-French pigtail catheter with a straightening obturator was placed over the wire and advanced. However, when the wire and obturator were removed, there was persistent pulsatile bleeding from the catheter. The overall chest tube placement procedure was performed without imaging guidance. A chest radiograph after chest tube placement showed the catheter tip projecting onto the cardiac silhouette with a persistent left pneumothorax (Fig.2). A subsequent non-contrast CT scan of the chest revealed the chest tube catheter tip inside the heart (Fig. 3, Fig. 4).

Although the patient was hemodynamically stable, an electrocardiogram revealed ST elevations secondary to cardiac injury. A limited echocardiogram further revealed a severely reduced ejection fraction of 28%. She was taken emergently to the operating room for sternotomy and mediastinal exploration, where the chest tube was found to have transected the diagonal coronary artery branch into the left ventricular cavity. After the malpositioned chest tube was removed, she underwent evacuation of the left pneumothorax and hemothorax, as well as repair of the left ventricular injury. Once hemostasis appeared to be adequate, a 28-French straight chest tube and 24-French Blake drain in the mediastinal space were placed. She was then admitted to the Cardiothoracic ICU on postoperative day 0, and the Surgical ICU on postoperative day 1. A persistent small apical left pneumothorax remained on postoperative day 5, likely secondary to apical scar tissue noted intraoperatively. By postoperative day 8, the patient’s pneumothorax had predominantly resolved, leaving only a stable, small residual pneumothorax. She was then downgraded to the floor. A subsequent echocardiogram showed moderate improvement, with an ejection fraction of 41%, likely a low baseline due to the patients’ chronic comorbidities. The mediastinal drain was removed on postoperative day 6, and chest tube was removed on postoperative day 13 uneventfully, with the patient making a significant recovery for his acute injuries.

At the time of discharge, given the evidence of chronic decompensated heart failure, the patient was placed on Furosemide, Hydralazine, Isordil, and Spironolactone, with further instructions to start an ACE inhibitor in their outpatient follow-up. However, chart review was notable for lack of follow-up with a primary care provider or cardiologist. As a result of the patient’s unhoused status, HIV/AIDS, and decompensated heart failure, history was notable for numerous visits to the ED in the subsequent five months following discharge. During these admissions, further evaluations, including multiple EKGs, chest radiographs, and chest CT scans, were unremarkable for serious complications related to potential long-term sequelae of the iatrogenic cardiac injury. Incidentally noted, however, were a small, chronic left-sided pleural effusion and minor bibasilar atelectasis, likely a result of their chronic stable HFrEF.

## Discussion

A retrospective review of patients undergoing tube thoracostomy over a 10-year period found complications in 20% of patients, with over 90% of these complications being positional with tube malfunction and serious complications being rare [1]. The overall median complication rate for tube thoracostomy has been determined to be 19% in a systematic review and meta-analysis, and complications can be categorized into insertional, positional, removal, infectious or malfunction complications [2]. However, accidental placement into the heart chambers remains extremely rare [[3], [4], [5], [6]].

Recognizing early warning signs of cardiac injury during chest tube placement, particularly in high-risk patients with significant comorbidities, is critical to preventing worsening decompensation in patients. Some of the prominent clinical signs include chest pain, hypotension, bleeding, tamponade, and arrhythmias, which should help prompt further workup including electrocardiogram, echocardiogram, and diagnostic imaging. As was the case with our patient, immediate imaging was a critical factor in reducing morbidity and mortality (Fig. 1, Fig. 2, Fig. 3, Fig. 4). Given the ubiquity of echocardiography, it is an indispensable diagnostic tool that can be used at the bedside to address a number of clinical questions in iatrogenic cardiac injury, including whether the patient has an acute hemopericardium or tamponade, their stability for transfer, what type of intervention is warranted, and whether the intervention was successful [3]. While the role of chest radiograph is still invaluable, given the lack of specificity for diagnosing cardiac injury [4], echocardiography is the recommended first-line diagnostic tool for this type of concern for cardiac injury.

Although not completely preventable, this case highlighted the necessity for mitigating complications during chest tube placement with the proper utilization of image guidance in the future. Imaging, such as with CT and ultrasound guidance, have been shown to provide a successful, secure method of percutaneous intrathoracic collection drainage as an alternative to surgical treatment [7]. Furthermore, ultrasound can safely and effectively locate the intercostal space for tube thoracostomy incision site, addressing yet another potential complication of chest tube placement [[8], [9]]. Imaging can also be utilized to avoid larger organ and vascular injuries, as well as quickly detect tube malposition [10].

## Conclusion

In conclusion, our case study involving an iatrogenic chest tube placement within a patient’s heart was a rare adverse occurrence that underscores the importance of careful pre-procedural planning, vigilant hemodynamic monitoring, and rapid response protocols. While this patient fortunately experienced significant recovery within 13 days without additional long-term complications, the incident emphasizes the critical role that timely recognition, appropriate imaging, and rapid intervention have in minimizing adverse events and optimizing outcomes. This patient’s experience has further reinforced the recommendation for performing chest tube placements under image guidance in order to help prevent the catastrophic adverse events, regardless of the additional potentially cumbersome or additional expenses associated with doing this procedure under image guidance.

## Patient consent

Written informed consent for the case report was obtained from the patient’s family (mother) due to the patient’s condition.
